# Effect of metformin monotherapy and dual or triple concomitant therapy with metformin on glycemic control and lipid profile management of patients with type 2 diabetes mellitus

**DOI:** 10.3389/fmed.2022.995944

**Published:** 2022-10-14

**Authors:** Yan-Yu Lin, Shuen-Fu Weng, Chung-Huei Hsu, Chen-Ling Huang, Yu-Pei Lin, Min-Chun Yeh, A-Young Han, Yu-Shan Hsieh

**Affiliations:** ^1^Division of Endocrinology and Metabolism, Department of Internal Medicine, Taipei Medical University Hospital, Taipei City, Taiwan; ^2^Division of Endocrinology and Metabolism, Department of Internal Medicine, School of Medicine, College of Medicine, Taipei Medical University, Taipei City, Taiwan; ^3^Department of Internal Medicine, Taipei Medical University Hospital, Taipei City, Taiwan; ^4^Department of Nursing, College of Life Science and Industry, Sunchon National University, Suncheon, South Korea; ^5^School of Nursing, National Taipei University of Nursing and Health Sciences, Taipei City, Taiwan

**Keywords:** type 2 diabetes mellitus, metformin, glycemic control, lipid profile, concomitant therapy

## Abstract

**Background:**

In this study, we aimed to compare the effects of metformin-based dual therapy versus triple therapy on glycemic control and lipid profile changes in Taiwanese patients with type 2 diabetes mellitus (T2DM).

**Methods:**

In total, 60 patients were eligible for participation in this study. Patients received at least 24 months of metformin monotherapy, dual therapy, or triple therapy with metformin plus linagliptin (a dipeptidyl peptidase 4 (DPP-4) inhibitor) or dapagliflozin (a sodium-glucose cotransporter-2 (SGLT2) inhibitor). Blood samples were collected from each patient, followed by evaluation of changes in their blood glucose control and lipid profile-related markers.

**Results:**

A combination of metformin and DPP4 and SGLT2 inhibitor therapy more effectively reduced low-density lipoprotein cholesterol (LDL-C) (*p* = 0.016) than metformin monotherapy. A combination of metformin and DPP4 and SGLT2 inhibitor therapy more effectively improved total cholesterol (Chol, *p* = 0.049) and high-density lipoprotein cholesterol (HDL-C) than metformin monotherapy (*p* = 0.037). Metformin plus linagliptin dual therapy was more effective than metformin monotherapy in reducing glycosylated hemoglobin (HbA1C, *p* = 0.011). Patients who received a combination of linagliptin and empagliflozin showed a significant reduction in their fasting blood glucose (*p* = 0.019), HbA1c (*p* = 0.036), and Chol (*p* = 0.010) compared with those who received linagliptin dual therapy. Furthermore, patients who received metformin plus dapagliflozin and saxagliptin showed significantly reduced Chol (*p* = 0.011) and LDL-C (*p* = 0.035) levels compared with those who received metformin plus dapagliflozin.

**Conclusion:**

In conclusion, dual therapy with metformin and linagliptin yields similar glycemic control ability to triple therapy. Among metformin combination triple therapy, triple therapy of empagliflozin and linagliptin might have a better glycemic control ability than dual therapy of linagliptin. Moreover, Triple therapy of dapagliflozin and saxagliptin might have a better lipid control ability than dual therapy of dapagliflozin.

## Introduction

Asia is considered the epicenter of the global epidemic of type 2 diabetes mellitus (T2DM) because of rapid changes in eating habits and lifestyle and the increasing rates of obesity in Asia (1). Many studies have reported the epidemic of T2DM in East Asia and the interethnic differences in genetics, pathophysiology, eating habits, and lifestyle factors between Asian regions and also between Asian and Western countries (2).

Type 2 diabetes mellitus is characterized by chronic hyperglycemia and disturbances of carbohydrate, lipid, and protein metabolism. To date, patients with T2DM who initially achieve glycemic control with a single oral antidiabetic medicine usually require additional agents for maintaining glycemic control due to the progressive nature of the disease (3).

Several studies have verified the correlation between blood glucose levels and serum lipid profiles (4). Thus, use of an appropriate and cost-effective medication for T2DM is recommended. Concomitant use of multiple medicines is often indicated in the management of diseases; however, more medicines might not necessarily be better. Polypharmacy could result in increased healthcare costs and risks of adverse drug events and medication non-adherence (5). Therefore, to compare and confirm the different effects of monotherapy, dual therapy and triple therapy on lipid profiles and glucose control is important under the premise of minimizing side effects.

Metformin is recommended as a first-line oral glucose-lowering medication by the American Diabetes Association (6). It is usually prescribed along with other antidiabetic drugs such as sodium-glucose cotransporter-2 (SGLT2) inhibitors and dipeptidyl peptidase 4 (DPP4) inhibitors to control blood glucose and lipid levels. However, few studies have evaluated the efficiency of different types of metformin-based therapies in glycemic control and lipid profile management and compared them with metformin-based dual (with an SGLT2 inhibitor or a DPP4 inhibitor) and triple therapy (metformin with an SGLT2 inhibitor and a DPP4 inhibitor) in Taiwanese patients. Therefore, in the present study, we aimed to compare the effects of different types of metformin-based therapies on glycemic control and lipid profile changes in Taiwanese patients with T2DM.

## Materials and methods

### Patients

This study was conducted at Taipei Medical University Hospital, Taiwan. Participants who visited the endocrinology outpatient department between October 2021 and March 2022 were screened for eligibility. Individuals were eligible if they were above 20 years of age; had T2DM; who received at least 24 months of metformin monotherapy or combination therapy with linagliptin (DPP4 inhibitor) or dapagliflozin (SGLT2 inhibitor); and were on regular follow-up for blood tests for glucose, glycosylated hemoglobin (HbA1c), and lipid profile. Patients were excluded if they were receiving insulin injections or lipid-lowering drugs, such as statin. Individuals with renal or hepatic dysfunction or failed blood glucose control were also ineligible.

**FIGURE 1:**
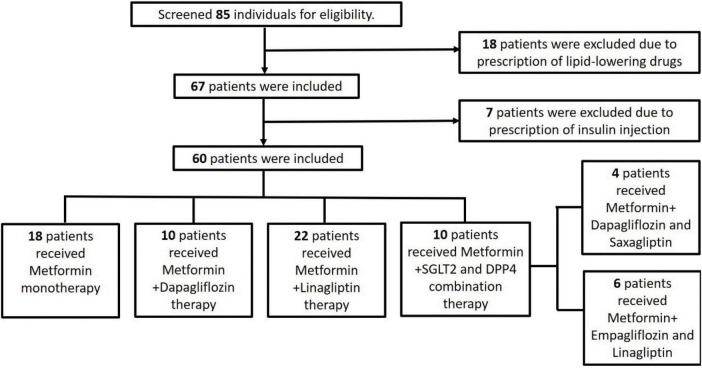
Flowchart showing patient inclusion in the study.

The study screened 85 individuals for eligibility. Of these patients, 18 patients were excluded, because they were prescribed lipid-lowering drugs, and 7 were excluded, because they were prescribed insulin injections. Figure 1 details the number of participants enrolled in the study along with the reason for exclusion of some participants. Finally, the study enrolled 60 participants whose demographic features have been summarized.

All patients provided their written informed consent prior to enrolment. The study protocol was approved by the ethics committee of the Institutional Review Board of Taipei Medical University (Approval No: N202107021). All procedures accorded with the ethical standards of the responsible committee on human experimentation (institutional and national) and with the Helsinki Declaration.

### Patient grouping and assignment

Patients were divided into four groups according to the treatment they received: metformin 1,000 mg monotherapy (metformin only group, *N* = 18); metformin 1,000 mg and linagliptin 5 mg combination therapy (+ DPP4 group, *N* = 22); metformin 1,000 mg and dapagliflozin 10 mg combination therapy (+ SGLT2 group, *N* = 10); and either metformin 1,000 mg plus empagliflozin 10 mg and linagliptin 5 mg or dapagliflozin 10 mg and saxagliptin 5 mg combination therapy (+ SGLT2 and DPP4 group, *N* = 10).

The 10 patients who received DPP-4 inhibitor and SGLT-2 inhibitor combination therapy (triple therapy) included 6 patients with empagliflozin 25mg and linagliptin 5 mg and 4 patients with dapagliflozin 10mg and saxagliptin 5mg.

### Medicinal compliance

Medication compliance was evaluated by the self-report of remain pill count. When patients come back to follow up, we calculated remain pills from self-report. And remain doses of medicine were record.

### Hematological analysis

Routine blood tests were performed at the clinical laboratory. Blood samples were collected from each patient after an overnight fast. Serum uric acid and ketone levels were determined using a one-touch self-metabolic marker monitoring analyzer (FORA MD-6; Fora Care, Taipei, Taiwan). Fasting glucose (glucose AC) was analyzed according to the hexokinase method (ADVIA Chemistry XPT System, Siemens, Berlin, Germany), and HbA1c was determined by high-performance liquid chromatography using an automatic analyzer (Bio-Rad Variant II Turbo 2.0 System, Hercules, California, USA). Serum lactate dehydrogenase (LDH), C- peptide, creatine (Cr), Alanine aminotransferase (ALT), total cholesterol (Chol), triglyceride (Tg), low-density lipoprotein cholesterol (LDL-C), and high-density lipoprotein cholesterol (HDL-C) levels were analyzed by enzymatic methods using an automatic analyzer (ADVIA Chemistry XPT). Serum insulin antibody (insulin Ab) was measured by immunoradiometric binding assay in an automatic analyzer (PerkinElmer CSBio, Santa Clara, California, USA).

### Modified homeostasis model assessment-insulin resistance index

Homeostasis model assessment-insulin resistance index is a simple and useful method for evaluating insulin resistance. Modified HOMA-IR was calculated using the following equation: 1.5 + fasting blood glucose × fasting C-peptide/2,800 (7, 8). A result above 1.9 indicated early insulin resistance, whereas a result above 2.9 indicated significant insulin resistance (7, 8).

### Statistical analysis

Statistical analysis and data management were performed using IBM SPSS Statistics 22 (IBM Corp., Armonk, NY, USA). Data are expressed as the mean and median. The Chi-square test was used for results of nominal scale. Data distributions were analyzed by Shapiro-Wilks test for normality. Non-parametric statistics, which are Mann–Whitney U test with Kruskal-Wallis Test were used to determine whether any between-group differences existed in the groups of patients, the *post hoc* test was using Dunn *post hoc* test. The Wilcoxon signed-rank test was used to analyze the within-group differences in the paired results of patients. A confidence interval of 95% was employed, and *p* <0.05 indicated statistical significance.

## Results

### Baseline characteristics and comparisons between study groups

A total of 60 participants with T2DM who received different medication therapies were included. The statistical results indicated no significant difference between the groups of patients who received different therapies in T2DM duration, body mass index (BMI), age, fasting blood glucose, HbA1c, LDH, and HOMA-IR score and levels of insulin Ab, C-peptide, uric acid, ketone, Cr, and ALT (Table 1).

**TABLE 1:** Between-group analysis of clinical characteristics of the patients with type 2 diabetes mellitus (T2DM).

		Metformin											
	Reference range	Metformin Only(N = 18)		+ SGLT2(N = 10)			+DPP4 (N = 22)			+ SGLT2 and DPP4 (N = 10)			Sig.
		MEAN	Median	MEAN	Median	*p*	MEAN	Median	*p*	MEAN	Median	*p*	
Male (%)	**−**	55.0	**−**	80.0	**−**	0.063	68.0	**−**	0.292	80.0	**−**	0.119	0.206
Duration of T2DM (Month)	**−**	68.9	73.3	70.5	81.0	0.849	62.9	74.3	0.486	69.8	68.0	0.457	0.864
BMI	**−**	27.2	27.3	30.9	28.5	0.156	27.9	27.1	0.909	27.0	27.1	0.855	0.440
Age	**−**	55.1	54.0	52.5	53.0	0.787	55.0	54.0	0.292	60.5	64.0	0.332	0.430
Glucose Ac	<100 mg/dl	172.1	139.0	185.3	156.5	0.641	145.9	146.0	0.537	176.7	173.5	0.291	0.301
HbA1c	<5.5%	7.9	7.2	7.6	6.8	0.723	7.2	6.7	0.389	7.4	7.3	0.614	0.667
LDH	98-192U/L	199.1	179.0	196.3	185.0	0.755	195.0	198.0	0.577	202.0	175.0	0.549	0.775
Insulin Ab	<7.5%	5.9	6.0	6.2	5.6	0.675	6.1	6.1	0.437	5.6	5.2	0.597	0.726
C-peptide	0.8-3.8ng/mL	2.8	2.5	3.7	3.5	0.113	3.2	3.1	0.800	2.7	2.3	0.684	0.256
HOMA-IR	<1.9	1.7	1.6	1.7	1.7	0.573	1.7	1.6	0.393	1.7	1.6	0.924	0.676
Uric acid	0.24-0.51 μmol/L	352.8	102.0	341.0	148.0	0.851	334.9	128.1	0.803	335.5	330.1	0.100	0.997
Ketone	< 0.6 mmol/L	0.7	0.7	0.6	0.7	0.904	1.0	0.5	0.281	0.5	0.5	0.406	0.445
Cr	0.7-1.2 mg/dl	0.9	0.9	0.9	0.8	0.884	1.5	1.0	0.127	0.9	0.9	0.563	0.404
ALT	< 40U/L	24.2	23.0	31.0	28.0	0.371	30.3	28.0	0.166	28.0	28.5	0.350	0.605

DPP-4: dipeptidyl peptidase-4 inhibitor; SGLT-2: sodium glucose transporter-2; BMI; LDH: lactate dehydrogenase; Glucose AC: fasting blood glucose; HbA1c: glycosylated hemoglobin; C-peptide: C-reactive peptide; insulin Ab: insulin antibodies; HOMA-IR: Homeostasis Model Assessment-Insulin Resistance Index; Cr: creatinine; ALT: Alanine Aminotransferase.

### Between-group analysis of changes in lipid profiles

A significant difference was observed in the lipid profiles of patients in the metformin only group and the + SGLT2 and DPP4 group in terms of Chol (*p* = 0.033), HDL-C (*p* = 0.011), and LDL-C levels (*p* = 0.015). As indicted in Table 2, significantly different changes were observed in serum Chol (*p* = 0.010) between the + SGLT2 and DPP4 group and the DPP4 group. Similarly, significantly different changes were observed in Chol (*p* = 0.002) and LDL-C (*p* = 0.008) levels between patients treated with + SGLT2 and DPP4, and SGLT2.

**TABLE 2:** Within-group analysis of glycemic control- and lipid profile-related biomarkers before and after treatment in each group.

		Metformin											
	Reference range	Metformin Only		+ SGLT2			+DPP4			+ SGLT2 and DPP4			Sig.
		MEAN	Median	MEAN	Median	*p*	MEAN	Median	*p*	MEAN	Median	*p*	
Glucose AC (B)	< 100mg/dl	200.9	117.1	235.2	164.5	0.092	290.3	23.3	0.448	239.9	95.0	0.266	0.708
Glucose AC		172.1	77.4	185.3	84.9	0.641	145.9	43.3	0.537	176.7	48.5	0.291	0.301
Δ		−28.8		−49.9			−144.4			-63.2			
*p*		0.064		0.297			0.009**			0.035*			
HbA1c (B)	< 5.5%	8.8	2.9	8.5	2.9	0.675	8.6	2.6	0.810	7.8	1.9	0.769	0.982
HbA1c		7.9	2.7	7.6	2.0	0.723	7.2	1.4	0.389	7.4	1.2	0.614	0.667
Δ		−0.9		−0.8			−1.4			−0.5			
*p*		0.077		0.233			0.042*			0.019*			
Chol (B)	< 5.2 mmol/L	5.6	5.3	5.1	5.3	0.100	4.5	4.7	0.900	4.3	4.6	0.085	0.123
Chol		4.2	4.2	4.5	4.5	0.378	4.3	3.7	0.842	3.3	3.2	0.033*	0.010*
Δ		−1.4		−0.6			−0.2			−1.1			
*p*		0.002**		0.114			0.044*			0.049*			
Tg (B)	< 1.69 mmol/L	2.4	2.0	1.8	1.7	0.306	2.1	1.6	0.418	1.7	1.7	0.308	0.699
Tg		1.6	1.4	1.7	1.7	0.535	1.9	1.2	0.433	1.2	1.0	0.792	0.197
Δ		−0.8		−0.1			−0.2			−0.5			
*p*		0.035*		0.812			0.169			0.037*			
HDL-C (B)	> 1.53 mmol/L	1.0	1.0	1.2	1.2	0.085	1.1	1.1	0.269	1.0	1.0	0.456	0.171
HDL-C		1.1	1.1	1.2	1.2	0.113	1.2	1.2	0.522	1.2	1.2	0.011*	0.461
Δ		0.1		0.0			0.0			0.1			
*p*		0.115		0.492			0.316			0.160			
LDL (B)	< 2.6 mmol/L	3.5	3.3	3.4	3.8	0.826	2.7	2.5	0.350	2.6	2.8	0.500	0.057
LDL-C		2.6	2.5	2.8	2.8	0.616	2.4	2.0	0.431	1.5	1.6	0.015*	0.016*
Δ		−0.8		−0.6			−0.3			−1.1			
*p*		0.002**		0.097			0.017*			0.176			

B: before treatment result; Glucose AC: fasting blood glucose; HbA1c: glycosylated hemoglobin; Chol: total cholesterol; Tg: triglyceride; LDL-C: low-density lipoprotein cholesterol; HDL-C: high-density lipoprotein cholesterol. Δ: difference between the before treatment result and recent result. *p < 0.05; **p < 0.01. Red values indicate significant differences.

### Within-group analysis of lipid profiles before and after treatment in each group

The metformin monotherapy group exhibited significantly reduced levels of GPT (*p* = 0.01), Chol (*p* = 0.01), and LDL-C (*p* = 0.001) after treatment. Moreover, the metformin combined with linagliptin (+ DPP4) group exhibited significantly reduced Glucose AC (*p* = 0.035) and HbA1c (*p* = 0.019) relative to the pretreatment treatment. Similarly, the + SGLT2 and DPP4 group exhibited significantly reduced fasting blood Glucose (*p* = 0.035), HbA1c (*p* = 0.019), GPT (*p* = 0.007), Chol (*p* = 0.035), and Tg (*p* = 0.045) levels relative to the pretreatment values. The changes in lipid profile are presented in Table 2.

### Effect of different combination therapies on lipid profile and blood glucose control

To further verify the effect of metformin plus empagliflozin and linagliptin and metformin plus dapagliflozin and saxagliptin in the + SGLT2 and DPP4 group, the groups of patients receiving metformin plus linagliptin and empagliflozin and those receiving metformin plus linagliptin treatment were analyzed. A significant difference was observed in the fasting blood *glucose* (*p* = 0.019) and HbA1c (*p* = 0.036) levels (Table 3) between patients receiving linagliptin and those receiving empagliflozin with linagliptin. Similarly, a significant difference was observed in Chol (*p* = 0.019) and LDL-C levels (*p* = 0.035) (Table 4) between patients who received dapagliflozin and saxagliptin.

**TABLE 3:** Effects of metformin with linagliptin dual therapy and metformin with linagliptin and empagliflozin triple therapy on lipid profile and blood glucose control of patients with type 2 diabetes mellitus.

		Metformin				
	Reference range	+ DPP4 (Linagliptin)		+SGLT2 and DPP4 (Empagliflozin and Linagliptin)		*p*
		MEAN	Median	MEAN	Median	
Duration of T2DM (Month)	**−**	62.9	66.8	58.35	68.00	0.783
Glucose AC (B)	< 100mg/dl	290.3	23.3	234.8	118.7	0.586
Glucose AC		145.9	43.3	128.7	31.50	0.019*
Δ		−144.4		−106.2		
*p*		0.009**		0.543		
HbA1c (B)	< 5.5%	8.6	2.6	7.8	1.93	0.487
HbA1c		7.2	1.4	6.7	0.91	0.036*
Δ		−1.4		−1.1		
*p*		0.042*		0.646		
Chol (B)	< 5.2 mmol/L	4.5	4.7	4.4	4.15	0.445
Chol		4.3	3.7	3.4	3.08	0.010*
Δ		−0.2		−1.0		
*p*		0.044*		0.014*		
Tg (B)	< 1.69 mmol/L	2.1	1.6	1.9	1.81	0.814
Tg		1.9	1.2	1.5	1.47	0.245
Δ		−0.2		−0.5		
*p*		0.2		0.619		
HDL-C (B)	> 1.53 mmol/L	1.1	1.1	0.9	0.96	0.112
HDL-C		1.2	1.2	1.2	1.1	0.847
Δ		0.1		0.3		
*p*		0.316		0.848		
LDL-C (B)	< 2.6 mmol/L	2.7	2.5	2.5	2.04	0.588
LDL-C		2.4	2.0	1.9	1.67	0.124
Δ		−0.3		−0.6		
*p*		0.017*		0.274		

BH: body height; BW: body weight; LDH: lactate dehydrogenase; Glucose AC: fasting blood glucose; HbA1c: glycosylated hemoglobin;Chol: total cholesterol; Tg: triglyceride; LDL-C: low-density lipoprotein cholesterol; HDL-C: high-density lipoprotein cholesterol. *p < 0.05, **p < 0.01. Red values indicate significant differences.

**TABLE 4:** Effects of metformin with dapagliflozin dual therapy and metformin with dapagliflozin and saxagliptin triple therapy on lipid profile and blood glucose control in patients with type 2 diabetes mellitus.

		Metformin				
	Reference range	+ SGLT2 (Dapagliflozin)		+SGLT2 and DPP4 (Dapagliflozin and Saxagliptin)		*p*
		MEAN	Median	MEAN	Median	
Duration of T2DM (Month)	**−**	70.46	76.2	74.7	74.7	0.542
Glucose AC (B)	< 100mg/dl	235.2	164.5	242.5	253.0	0.933
Glucose AC		185.3	84.9	182.5	24.4	0.888
Δ		−49.9		−60.0		
*p*		0.297		0.984		
HbA1c (B)	< 5.5%	8.5	2.9	9.1	8.9	0.656
HbA1c		7.6	2.0	8.4	8.30	0.963
Δ		−0.8		−0.7		
*p*		0.233		0.900		
Chol (B)	< 5.2 mmol/L	5.1	5.3	4.7	4.87	0.336
Chol		4.5	4.5	3.3	3.28	0.011*
Δ		−0.6		−1.4		
*p*		0.114		0.309		
Tg (B)	< 1.69 mmol/L	1.8	1.7	1.4	1.27	0.520
Tg		1.7	1.7	1.2	0.90	0.133
Δ		−0.1		−0.2		
*p*		0.812		0.824		
HDL-C (B)	> 1.53 mmol/L	1.2	1.2	1.1	1.00	0.817
HDL-C		1.2	1.2	1.1	1.10	0.606
Δ		0.0		0.0		
*p*		0.492		0.168		
LDL-C (B)	<2.6 mmol/L	3.4	3.8	2.8	3.10	0.354
LDL-C		2.8	2.8	1.5	1.70	0.035*
Δ		−0.6		−1.3		
*p*		0.097		0.120		

Glucose AC: fasting blood glucose; HbA1c: glycosylated hemoglobin; Chol: total cholesterol; Tg: triglyceride; LDL-C: low-density lipoprotein cholesterol; HDL-C: high-density lipoprotein cholesterol. *p < 0.05. Red values indicate significant differences.

### Medicinal compliance

When calculated remain pills from self-report (since last 3 months), mean remain dose by drug was not statistically different each group. The remain medicine dose of groups were 1.9 (median: 2.0), 1.0 (median: 1.0), 2.0 (median: 2.5) and 2.4 (median: 2.0) doses (*p* = 0.132), respectively (data not shown).

## Discussion

The main finding of this study is that although a combination of metformin with a DPP4 and an SGLT2 inhibitor more effectively improved Chol and LDL-C than a combination of metformin and linagliptin or dapagliflozin dual therapy did. However, a similar effect also was found on reducing fasting glucose and HbA1C levels with metformin and linagliptin dual therapy.

Although the reasons for high or low medication adherence differ among patients, complicated regimens and a larger number of medications can reduce compliance (9). Compliance to therapy is important point in chronic conditions. Methods of measuring adherence can be either direct (biological marker) or indirect (self-reporting, questionnaires, pill counts) (10). Therefore, we calculated the remain dose of pills when every return visit for ensure homogeneity of study population. Base on the remain pill counts form study population by self-report, we ensured treatment compliance homogeneity of study population in groups.

Metformin is the first-line treatment for individuals with newly diagnosed T2DM. Our study corroborated previous findings that metformin monotherapy considerably improves dyslipidemia in statin-naive individuals with T2DM (11), especially by reducing serum LDL-C *via* an AMP-activated protein kinase pathway (12). Metformin has been reported to improve insulin sensitivity by increasing it; it also reduces the rate of lipolysis, thereby decreasing the conversion of free fatty acids in the liver (13). A previous meta-analysis showed that metformin reduced body weight and improved the lipid profiles of 60-year-old participants. The results also suggest that metformin treatment may reduce the risk of major coronary events and all-cause mortality in diabetic populations (14).

The mechanism through which DPP4 inhibitors affect the lipid profile in T2DM remains poorly understood. This effect could be explained by glucagon-like peptide-1 receptor. DPP4 inhibitors might inhibit lipid absorption in the gastrointestinal tract. The action of DPP4 inhibitors is based on their prevention of the inactivation of incretin. A previous study reported that these compounds improve glycemic control, both when applied in monotherapy and in combination with other oral hyperglycemic agents. Patients with different levels of glycemic control who received DPP4 inhibitors combined with metformin therapy exhibited reduced fasting blood glucose and HbA1C levels compared with those who received continuous therapy with metformin alone (15). This result is consistent with our findings in presented study.

Previous findings have indicated that individuals from East Asia have lower insulin resistance and greater sensitivity to the incretin effect (16). In particular, the glucose control efficacy of DPP4 inhibitors or incretin receptor agonists has been reported to be greater in Asian populations, especially Japanese (17) and Korean populations (18). The difference in the treatment responses could be ascribed to a different lower insulin secretory function and less insulin resistance in T2DM or the different genetics in these populations. Previous results show that DPP4 inhibitors achieve good glucose control in the Asian population (19). This result is consistent with our findings in a Taiwanese population. However, in our study, the triple therapy group also showed good glucose control and lipid profile effects.

Dyslipidemia is associated with an increased risk of cardiovascular disease in patients with T2DM. Previous studies have also suggested that the lipid-reducing efficiency of linagliptin or metformin may be lower than that of dapagliflozin (20) and a placebo (21). The different effects of monotherapy, dual therapy, and triple therapy with metformin on the lipid profiles of patients with T2DM in this study are largely consistent with the results of previous studies (22–24). In our study, triple therapy reduced the lipid profile as effectively as linagliptin dual therapy did, there were also significant differences when compared with metformin monotherapy. However, the mean Chol, HDL-C, and LDL-C levels significantly differed from baseline to after treatment DPP4 and SGLT2 inhibitor combination therapy (62.9 months on average).

In terms of blood glucose control, early combination treatment with linagliptin and metformin has been reported to improve hyperglycemia, resulting in a significant reduction in fasting glucose relative to that achieved after metformin monotherapy (25). Another study reported that compared with linagliptin monotherapy, linagliptin plus metformin treatment significantly reduced HbA1c levels after 24 weeks relative to the baseline levels (26). According to present results, similar to triple therapy, linagliptin dual therapy could improve blood glucose and HbA1C levels.

Previous studies reported that combination therapy with empagliflozin, linagliptin, and metformin (22) or dapagliflozin, saxagliptin, and metformin (27) produced considerable glucose-lowering effects in patients with T2DM. In a 52-week study, reductions in HbA1c with empagliflozin plus linagliptin therapy were superior to those with the joint use of either empagliflozin or linagliptin with metformin (28). To further verify this effect, we classified patients in the metformin triple therapy group into two different groups and reanalyzed the findings. We found that the addition of empagliflozin to metformin plus linagliptin led to a more effective reduction in fasting glucose, HbA1C l and Chol levels.

Moreover, the addition of saxagliptin to metformin plus dapagliflozin therapy led to a more effective reduction in serum Chol and LDL-C levels (Table 4). The results may indicate that a combination of metformin plus both linagliptin and saxagliptin affects the lipid profile in a manner different from that observed when a combination of metformin plus both empagliflozin and dapagliflozin is used. In other words, our results suggest that empagliflozin might have a better glycemic control ability than dapagliflozin. The results showed improvement in fasting glucose and HbA1C levels similar to that in a previous study (29) and demonstrated that saxagliptin might afford better LDL-C control than linagliptin. In the results of previous cross-sectional study, saxagliptin users had a significantly lower CVD risk than other DPP-4 drug users matched for sex, age, duration of drug use, systolic blood pressure, lipid profile, and fasting glucose (30). However, further large-scale observational studies evaluating the differences among these drugs is in terms of their cardiovascular benefits or glucose control abilities are needed.

This study has some limitations. First, our study was conducted at a single center, and the sample size was relatively small. Second, owing to the retrospective nature of the study, the medication history of the participants was not controlled, and whether the participants had ever been prescribed other antidiabetic medicines with varying drugs was unclear. Therefore, the interaction effects or side effects of the drugs may have been underestimated. Third, because this study only involved Taiwanese people, ethnic differences could not be accounted for. Thus, we followed a strict patient selection protocol to ensure homogeneity between groups. Further studies are needed to apply the results of this study to larger populations.

## Conclusion

In conclusion, we report that dual therapy with metformin and linagliptin yields similar glycemic control ability to triple therapy. Among metformin combination triple therapy, triple therapy of empagliflozin and linagliptin might have a better glycemic control ability than dual therapy of linagliptin. Moreover, Triple therapy of dapagliflozin and saxagliptin might have a better lipid control ability than dual therapy of dapagliflozin.

Combination therapy of metformin with an SGLT2 inhibitor and a DPP4 inhibitor may be an effective, but albeit relatively expensive, treatment for patients with T2DM. Thus, based on the results, dual therapy with metformin and linagliptin may be a better option for long-term glycemic control because of the similar glucose control ability to triple therapy. Further studies should investigate the long-term efficacy and cost-effectiveness of each combination therapy. These results could provide a guide for clinical physicians to select a more appropriate prescription from metformin monotherapy, dual therapy, or triple therapy in the future.

## Data availability statement

The original contributions presented in this study are included in the article/supplementary material, further inquiries can be directed to the corresponding author/s.

## Ethics statement

The studies involving human participants were reviewed and approved by Research Ethics Committee of Institutional Review Board of Taipei Medical University (Approval No: N202107021). The patients/participants provided their written informed consent to participate in this study.

## Author contributions

Y-SH and Y-YL designed this study, collected and analyzed the data. Y-SH, Y-YL, and S-FW wrote the main manuscript. C-HH, C-LH, and Y-PL revised the manuscript. M-CY and A-YH reviewed the manuscript and provided the recommend in study design. All authors reviewed the manuscript.
